# Perianal Paget’s Disease From Underlying Anal Adenocarcinoma: A Case Study

**DOI:** 10.7759/cureus.99809

**Published:** 2025-12-22

**Authors:** Christina L Van Hemmen Kon, Lauren Brick, Briana Valli, David Marshall, Jeffrey Snow, Jordan J Ditchek, Gary Schwartz

**Affiliations:** 1 Medical Education, Nova Southeastern University Dr. Kiran C. Patel College of Allopathic Medicine, Fort Lauderdale, USA; 2 Pathology, Memorial Healthcare System, Hollywood, USA; 3 General Surgery, Memorial Healthcare System, Hollywood, USA; 4 Radiology, Memorial Healthcare System, Hollywood, USA

**Keywords:** anal adenocarcinoma, chemoradiotherapy, cytokeratin 20, extramammary paget disease, immunohistochemistry, metastatic adenocarcinoma, mucinous adenocarcinoma, perianal neoplasms, perianal paget disease, secondary paget disease

## Abstract

Perianal Paget's disease (PPD) is a rare intraepithelial adenocarcinoma with epidermatotropic features, which often presents with common dermatological conditions such as eczema, hemorrhoids, and sensations of itching and burning. Due to these nonspecific symptoms and limited data related to this rare disease, its diagnosis is often delayed. However, as this disease is often secondary to an underlying malignancy, its early diagnosis may prevent fatal outcomes.

This case report discusses a 76-year-old female patient with a family history of colon cancer who presented with anal pain, bleeding hemorrhoids, and a perianal mass. The initial lesion was removed using transanal excision surgery. Several months later, a PET/CT scan showed hyperintense lymph nodes in the right external iliac vessels; upon biopsy, metastatic anal adenocarcinoma was diagnosed. The patient underwent subsequent systemic chemotherapy and chemoradiation.

This case emphasizes the diagnostic pitfalls in the setting of coexisting pelvic pathology and the importance of early detection, recognition of variance in immunohistochemical markers, and timely interventions in PPD for the most efficacious treatment. It also highlights the need to determine the source of PPD and to increase physician awareness of secondary PPD arising from anal adenocarcinoma.

## Introduction

Perianal Paget's disease (PPD) is a rare subtype of extramammary Paget's disease (EMPD) featuring an often-insidious clinical presentation, with pruritus, erythema, rashes, and paresthesia being the most common symptoms. Due to its indolent course and non-specific symptoms, many patients are initially treated for benign conditions such as eczema or hemorrhoids. Therefore, patients with PPD often do not receive the proper diagnosis until these treatments are deemed refractory [1].  

 Diagnosis of PPD requires both histopathology and immunohistochemical (IHC) analysis to distinguish primary EMPD from secondary EMPD, which is often gastrointestinal in nature [2]. Due to a lack of clinical understanding and limitations in clinician familiarity, there are inconsistencies in diagnostic approach, leading to misdiagnosis and diagnostic delays. These misdiagnoses and delays are further exacerbated when there are coexisting pathologies that add to the diagnostic complexity, including concurrent fibroids, pelvic masses, and lymphadenopathies. Furthermore, due to its low prevalence, standardized treatment strategies for PPD are not yet defined. In localized lesions, wide local excision is generally preferred [3]. Other therapies such as photodynamic therapy, Mohs surgery, and topical antineoplastics are also considered [4]. Secondary PPD often requires systemic management due to the underlying malignancy, facilitating regression of the Paget’s lesion.  

This case report helps expand the current literature regarding PPD and provides a unique case presentation to increase the standardization and effectiveness of the diagnosis and treatment of this rare disease. 

## Case presentation

**Figure 1:**
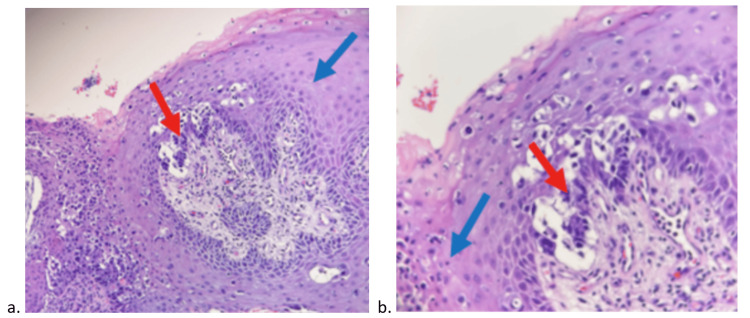
Histopathologic findings H&E staining of the tumor in (a) low power and (b) high power, demonstrating the Paget cells (red arrows) percolating through the epidermis (blue arrows).

A 76-year-old woman with a history of hemorrhoids and diverticulosis and a family history of colon cancer presented with anal pain, bleeding hemorrhoids, and "something strange next to her anus." Upon physical examination of the anus, pink colored, dome-shaped bumps at the three o’clock position were noted. A biopsy obtained via colonoscopy tested positive for adenocarcinoma with mucinous features, moderately differentiated with pagetoid spread to the overlying squamous epithelium (Figure 1).   

**Figure 2:**
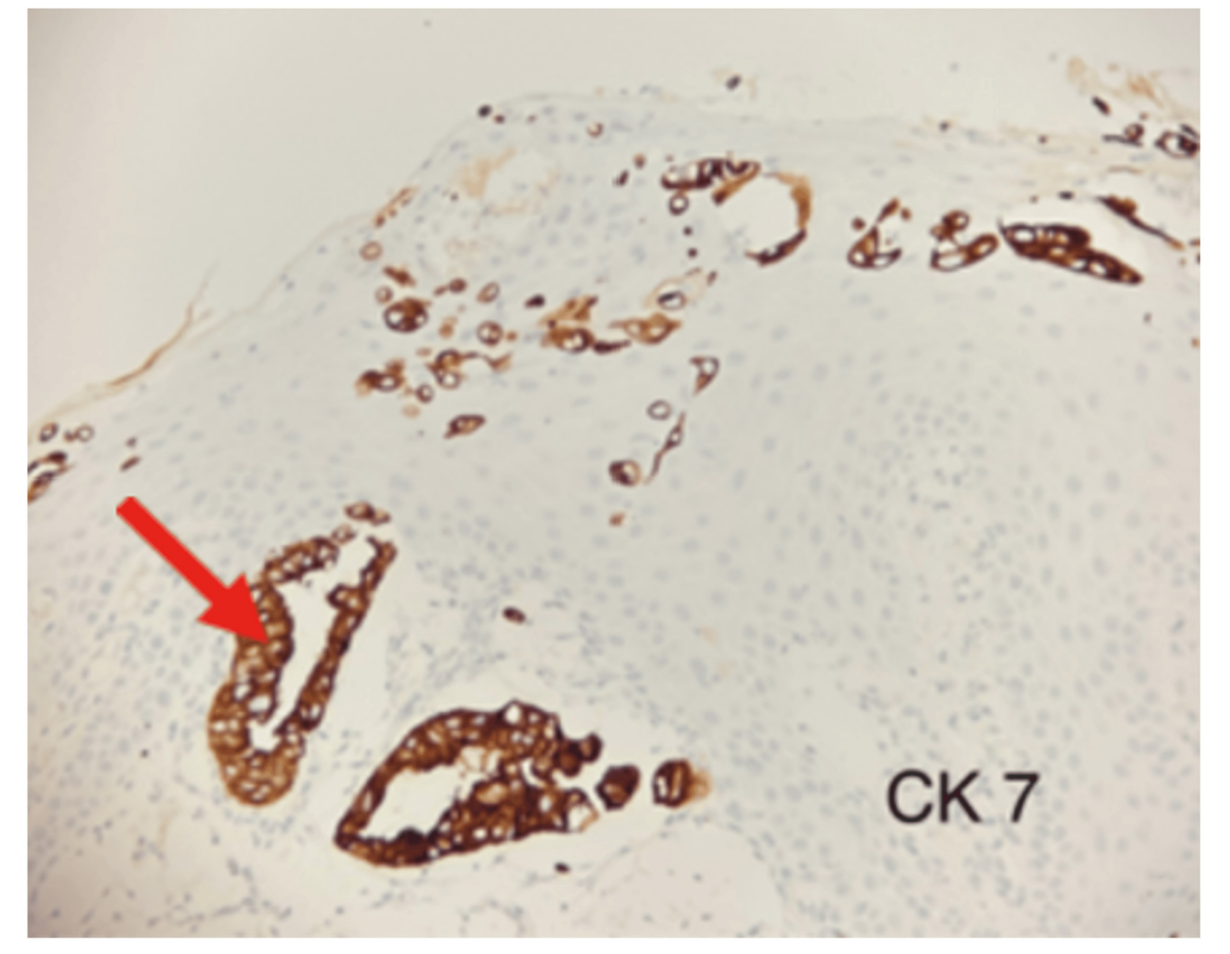
CK-7 stain The CK-7 stain demonstrates the tumor/Paget’s cells (red arrow); the squamous epithelium does not stain.

Histopathological examination indicated that the neoplasm is positive for cytokeratin 7 (CK7) and cytokeratin 20 (CK20), and negative for tumor protein 63 (p63) (Figure 2).  

A PET/CT scan was conducted, indicating no metastasis; however, it did expose a retroverted midline uterine mass. Transanal tumor excision was performed using an elliptical incision with a 2mm margin around the lesion. The sphincter muscle was preserved, and a biopsy was sent to pathology for analysis of the distal margins. There were no complications during the procedure or during the postoperative period.    

Transvaginal ultrasound assessing the midline retroverted uterine mass confirmed the presence of an 8.8 x 1.9 cm pedunculated fibroid with calcifications. As there was increased uptake noted on the PET/CT, the patient was referred to gynecology and oncology for monitoring of possible malignant transformation to leiomyosarcoma.    

Nine months after excision surgery, during a physical exam, a posterior non-tender uterine mass and right inguinal lymphadenopathy were noted. Subsequent MRI of the pelvis with and without contrast noted a heterogeneous solid cystic lesion in the pouch of Douglas, consistent with earlier considerations of fibroids. However, since mildly enlarged T2 hyperintense lymph nodes were present along the external iliac vessels, with the largest measuring 2.8 x 1.8 cm, malignancy could not be completely ruled out.    

**Figure 3:**
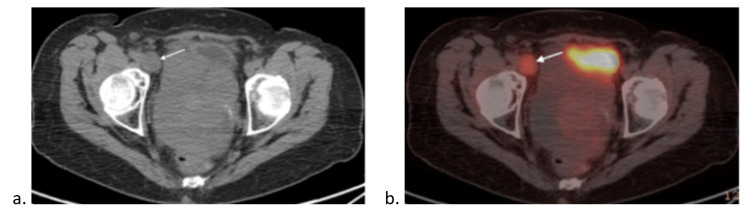
(a) PET scan and (b) CT scan demonstrating a hypermetabolic right external iliac node (white arrows)

**Figure 4:**
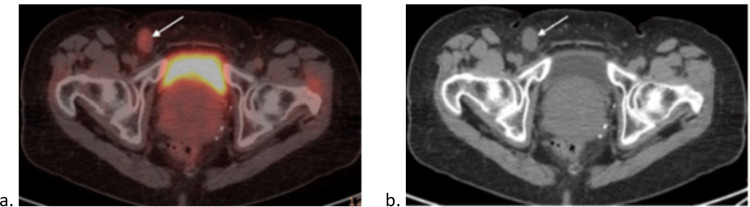
(a) PET Scan and (b) CT scan demonstrating a hypermetabolic right inguinal node (white arrows)

A month later, a PET/CT demonstrated a hypermetabolic right external iliac node measuring 2.4 x 2.1 cm (Figure 3) and a hypermetabolic right inguinal node measuring 0.5 x 1.7 cm (Figure 4).   

**Figure 5:**
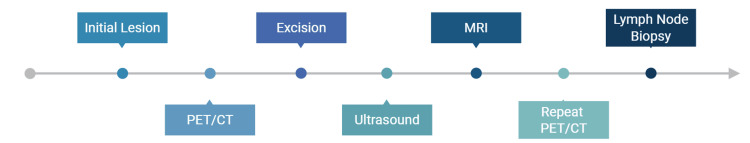
Sequence of diagnostic steps performed during this case.

There was improved non-specific anal uptake, and an unchanged bulky uterus with increased posterior uptake consistent with earlier evaluation of fibroids. The right external iliac lymph node biopsy showed metastatic adenocarcinoma with intestinal differentiation, most consistent with lower GI origin. Immunohistochemical analysis shows that the malignancy was positive for CK20, CDX2, villin, SATB2, and negative for PAX8 and GATA3. Labs showed elevated tumor markers CA19-9 (362), CEA (19.8), and CA125 (123) but were otherwise unremarkable. As the biopsy showed positive CK7, CK20, and negative p63, combined with the presentation of pagetoid spread, the pathology was deemed likely due to secondary EMPD [5]. These results, combined with the positive CDX2, villin, and SATB2 found in the lymph node, supported the diagnosis of metastatic anal adenocarcinoma, with the pagetoid spread being a cutaneous extension of the adenocarcinoma. The overall timeline of the diagnostic tests and procedures performed to reach this conclusion can be found in Figure 5. 

Management and treatment

After discussion of this case at the tumor board, the decision was made to start the patient on chemotherapy, particularly the mFOLFOX6 regimen, which contains leucovorin, 5-fluorouracil, and oxaliplatin, which are commonly used in the treatment of colorectal cancer [6]. After completion of the chemotherapy regimen, chemoradiation therapy (CXRT) was initiated as per the treatment protocol for colorectal cancer. Evaluation of the response to completion of the mFOLFOX-6 regimen and CXRT guided subsequent steps, including definitive salvage surgery in the case of present residual disease.    

The patient tolerated the mFOLFOX6 infusions well without major complications. After the second infusion, she reported a number of acute neuropathic side effects from the oxaliplatin, such as mild jaw cramping, slurred speech, and hypersensitivity to cold. She also experienced alopecia secondary to the cytotoxic drugs, as well as intermittent vaginal bleeding and epistaxis. An endometrial biopsy was performed, which was negative for malignancy. Additionally, she experienced mild anemia and thrombocytopenia, which resolved upon discontinuation of mFOLFOX-6.    

After completion of eight cycles of mFOLFOX-6, another PET/CT was conducted, showing stable right external iliac (2.1 cm, standardized uptake value (SUV) 2.2) and inguinal (2.1 cm, SUV 1.7) adenopathy with decreased metabolic activity, stable nonspecific anal canal uptake, and a persistent bulky fibroid uterus with heterogeneous uptake. She was scheduled for hysteroscopy and dilatation and curettage (D&C) upon completion of chemotherapy to assess the uterine mass. Following the procedure, she started final chemoradiation therapy with a dose of 50.4-54 gray in 28-30 daily fractions to the primary anal canal and regional lymph node combined with the 5-FU prodrug capecitabine in an attempt to achieve remission.    

## Discussion

PPD is a rare subtype of EMPD that presents most commonly in women in the sixth to eighth decades of life [7]. EMPD typically arises in the vulva, but can occur in the perineum, perianal region, scrotum, or penis [8]. Since it often presents similarly to common dermatological conditions such as eczema, hemorrhoids, and general sensations of pruritus or paresthesia, clinicians should consider PPD and perform a biopsy in patients who do not respond to initial treatment. In the case of a primary tumor, incidental deep invasion of the epithelium can occur. Perianal involvement is an uncommon presentation of EMPD and is often associated with an underlying malignancy, making early detection vital [8].   

**Figure 6:**
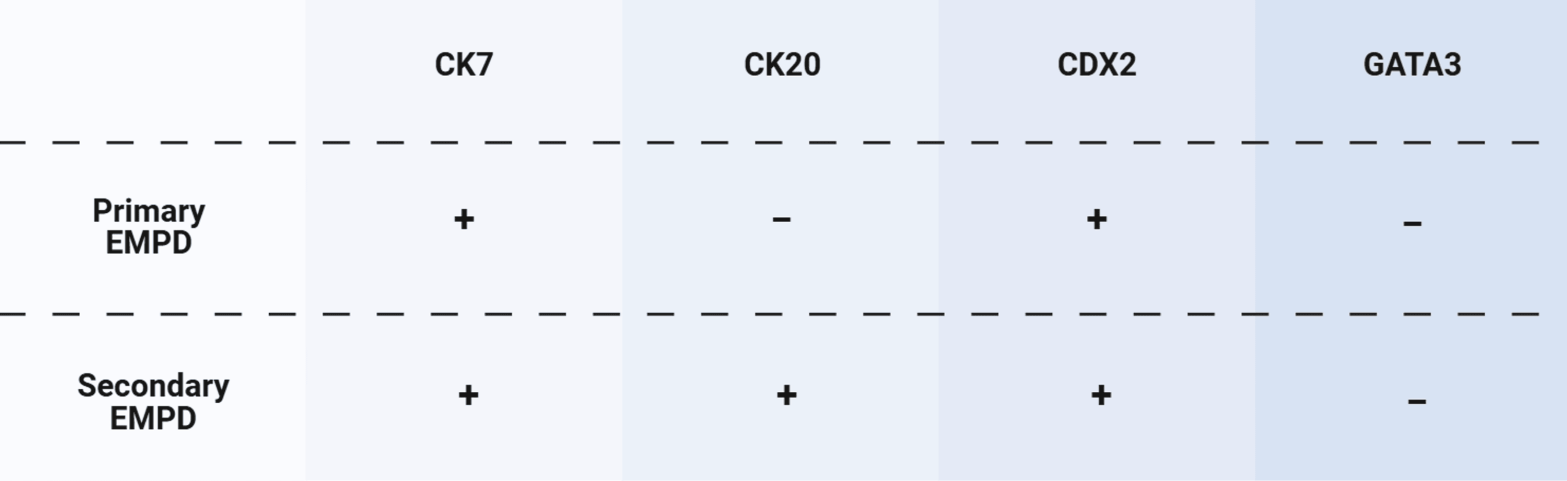
Differentiation of IHC markers for primary and secondary EMPD. IHC: immunohistochemical; EMPD: extramammary Paget's disease

The diagnosis of EMPD can be challenging due to its inconspicuous onset [9]. In this case study, the patient's history of hemorrhoids and anal irritation mimics other case series in which anal skin lesions are the first presentation of PPD. Differentiation between primary EMPD, cutaneous adenocarcinoma from apocrine or eccrine glands, as well as secondary EMPD, and epidermal extension of visceral malignancy was essential to determine the proper treatment. Prior studies emphasize this distinction, as secondary EMPD is frequently associated with underlying colorectal or urogenital carcinomas and requires an oncologic workup and management therapy, where primary disease can be managed with local approaches [9]. While older literature suggests that 12-14% of PPD manifestations were due to underlying gastrointestinal malignancies, recent research may suggest differently [3,6]. In a literature review of 159 cases of PPD spanning from 1996 to 2021, 70 of the PPD cases were primary in nature, and 89 were secondary to an underlying malignancy. This same study later included additional studies reinforcing this trend, with the majority being secondary forms of PPD. These findings indicate that secondary PPD is more common than previously appreciated, highlighting the importance of a comprehensive workup [10]. In this case, the lesion was determined to be secondary in nature. Despite having mucinous features and pagetoid spread, it lacked the tumor marker GATA3 and expressed CK20 and CDX2, which is atypical in primary EMPD presentation (Figure 6).

Additional immunohistochemical analysis showed positive staining for the villin and SATB2 markers, which, in combination with the patient’s family history, allowed for the determination of the lesion’s gastrointestinal origin. This case’s complex presentation delayed the exact diagnosis and initiation of treatment until after discussion by the GI tumor board.    

Coexisting lesions frequently confound metastatic attribution in PPD; thus, performing IHC and determining the nature of other existing pathologies is crucial [11]. In this case, the presence of pelvic pathologies further complicated the determination of metastasis origin and severity. Imaging showed uterine fibroids, a mass in the pouch of Douglas, as well as right external iliac lymphadenopathy, which has been associated with a worse prognosis in cases of PPD [12]. Due to the elevated CA-125 marker, the solid cystic lesion in the pouch of Douglas raised suspicion for a primary tumor of ovarian origin [9]. Alternatively, metastasis from the primary anal adenocarcinoma could explain the lesion, with resultant elevation in CA-125 from peritoneal inflammation. Biopsy and immunohistochemical analysis were needed to determine the origin of the tumor. Positive CK20, CDX2, villin, and SATB2 confirmed adenocarcinoma with intestinal differentiation, while PAX8 negativity ruled out ovarian malignancy, concluding that the lymphadenopathy was likely due to metastasis of anal adenocarcinoma. The elevation in CA-125 was likely due to peritoneal irritation or the metastasis of GI origin. The combination of benign and malignant pathologies complicated the determination of the origin of the lesion in the pouch of Douglas. This resulted in some uncertainty of the degree of metastasis and possible presence of a secondary primary tumor, increasing the difficulty of staging the malignancy and planning appropriate treatment. Incorrect allocation of the primary tumor’s origin can lead to inadequate treatment measures, such as gynecological surgery for ovarian masses versus treating the underlying anal adenocarcinoma.    

Management strategies differ markedly between primary and secondary PPD. In cases of primary PPD, where the lesion is confined to the epidermis, the gold standard of treatment is wide local resection [13]. In contrast, treatment for secondary PPD is often directed at treating the underlying malignancy with chemotherapy and radiotherapy, with the anticipation that the epidermal lesion regresses [14]. Current research shows the possibility of more targeted therapies, such as utilizing CO2 lasers for symptom management and hematoporphyrin-photodynamic therapy as a less invasive alternative to surgical excision [15].    

FOLFOX-6 is a chemotherapy regimen often used in colorectal cancer and comprises leucovorin, 5-fluorouracil, and oxaliplatin. There is evidence of successfully utilizing the regimen for perianal Paget's disease, where a 76-year-old woman with stage three EMPD was treated using FOLFOX, obtaining remission [15]. In the original FOLFOX-6 regimen, 5-fluorouracil is given as a bolus injection, followed by continuous infusion. However, mFOLFOX-6 is more commonly used, as it does not utilize the initial bolus of 5-fluorouracil due to hematologic toxicity such as neutropenia found in many patients. As mFOLFOX-6 has similar efficacy to the original FOLFOX-6, it is the preferred treatment in cases of colorectal cancer [16].   

Capecitabine is an oral prodrug of 5-fluorouracil and is commonly utilized as maintenance therapy after completion of mFOLFOX-6. Capecitabine halts the cell cycle in the S phase, which is more sensitive to radiation, making the treatment more effective [17]. Final chemoradiation therapy with a dose of 50.4-54 gray in 28-30 daily fractions is often utilized either neoadjuvant or as definitive treatment in non-operative cases [15,18]. Surveillance of recurrence should occur by physical exam quarterly throughout the first three years, and biannually up until five years after completion of treatment. Additionally, CT scans of the abdomen and pelvis are recommended quarterly throughout the first year and biannually upon completion of treatment. 

The five-year recurrence rate of PPD is approximately 40-61% following wide local resection, underscoring the challenges of achieving long-term disease control for this condition [19]. Due to the scarcity of available data, the recurrence rate when utilizing systemic chemotherapy remains unknown. There is some evidence that the prognosis of secondary PPD is similar to that of the underlying primary malignancy; however, this remains largely unknown [20]. The reported five-year survival rate is found to be favorable at 75-95% after five years [8,21]. Therefore, long-term patient surveillance should be based upon clinical indication [21]. Educating patients on self-examinations of the affected skin, recognition of symptoms, and early reporting to their physician may help improve early detection of recurrence.     

This case report is limited by its single-patient nature, which limits its generalizability. However, its findings reinforce what has been published in prior studies, suggesting that multidisciplinary care and close surveillance are vital due to the risk of underlying primary malignancies. This case aligns with the literature, highlighting the diagnostic and therapeutic challenges for this rare entity.   

## Conclusions

This case of secondary PPD arising from anal adenocarcinoma exemplifies the importance of early recognition and multidisciplinary care. Nonspecific, benign-appearing symptoms can cause misdiagnosis and delayed intervention. In this patient, prompt histopathological analysis and immunohistochemical profiling facilitated an accurate diagnosis and treatment plan, involving surgical excision, systemic chemotherapy with mFOLFOX-6, followed by chemoradiation. 

This case adds to the growing body of literature identifying secondary PPD associated with gastrointestinal malignancies, indicating that these cases could be more common than previously recognized. This supports the importance of performing timely biopsies of persistent or atypical perianal lesions in older adults and a comprehensive oncological workup. Early detection, appropriate IHC panels, and multidisciplinary care are crucial to the treatment and management of secondary PPD. 
